# The Fatigue Associated with Depression Questionnaire (FAsD): responsiveness and responder definition

**DOI:** 10.1007/s11136-012-0142-6

**Published:** 2012-03-09

**Authors:** Louis S. Matza, Kathleen W. Wyrwich, Glenn A. Phillips, Lindsey T. Murray, Karen G. Malley, Dennis A. Revicki

**Affiliations:** 1 Outcomes Research, United BioSource Corporation, 7101 Wisconsin Avenue, Suite 600, Bethesda, MD 20814 USA; 2 Eli Lilly and Company, Indianapolis, IN USA; 3 Malley Research Programming, Inc., Rockville, MD USA

**Keywords:** Fatigue, Depression, Responsiveness, Responder definition, Minimal important difference, Questionnaire

## Abstract

**Purpose:**

The Fatigue Associated with Depression Questionnaire (FAsD) was developed to assess fatigue and its impact among patients with depression. The purpose of this study was to examine the questionnaire’s responsiveness to change and identify a responder definition for interpretation of treatment-related changes.

**Methods:**

Data were collected at baseline and at 6 weeks from patients with depression starting treatment with a new antidepressant.

**Results:**

Of the 96 participants, 55.2% were women, with a mean age of 43.4 years. The total score and both subscales demonstrated statistically significant change with moderate to large effect sizes (absolute values ≥0.76). FAsD change scores were significantly correlated with change on the Brief Fatigue Inventory (*r* ≥ 0.73; *p* < 0.001). FAsD mean change scores discriminated among patient subgroups differing by degree of improvement in patient- and clinician-reported fatigue and depression. Responder definition for the two subscales and total score (0.67, 0.57, 0.62) was estimated primarily based on mean change among patients who reported a small but important improvement in fatigue.

**Discussion:**

The FAsD was responsive to change, and the responder definition may be used when interpreting treatment-related change. Results add to previous findings suggesting the FAsD is a useful measure of fatigue among patients with depression.

## Introduction

Research on treatment of depression has increasingly focused on a symptom-specific approach, often targeting residual symptoms that persist after other symptoms have improved [1–6]. Residual symptoms have been shown to predict relapse of depressive episodes [4, 7–10], and they contribute to functional impairment even after other depressive symptoms have improved following pharmacological or psychological treatment [8, 9, 11–13]. Much of the research on residual symptoms has focused on fatigue, which is one of the most common symptoms of major depressive disorder [14–16]. Several studies suggest that fatigue is frequently a residual symptom, persisting in roughly 20 to 38% of patients who have remitted following pharmacological treatment or psychotherapy [9, 17, 18]. There is a substantial and growing body of research focusing on fatigue associated with depression because of its prevalence, its resistance to treatment, and its association with impairment in social and work functioning [19–23].

Despite the clinical importance of fatigue associated with depression, there was no available patient-reported outcome (PRO) instrument designed specifically to assess fatigue and its impact among patients with depression [24]. Therefore, the Fatigue Associated with Depression Questionnaire (FAsD) was recently developed to address this gap in assessment tools for patients with depression [25]. Depression symptom measures often include an item assessing fatigue [26–28], but they do not provide a thorough multidimensional assessment of this construct, and they are therefore unlikely to adequately capture fatigue and its impact. In focus groups conducted when drafting the FAsD, patients reported a range of the 13 items of the FAsD were designed to capture a more thorough spectrum of fatigue experience and impact that is important to patients with depression.

Generic instruments, designed to be completed by respondents regardless of medical or psychiatric condition, are available for a more detailed assessment of fatigue [29, 30]. However, there is growing awareness that PRO instruments must demonstrate content validity and good measurement properties in the specific target population in order to be appropriate for assessment of treatment outcomes [31, 32], and the generic fatigue measures do not meet these standards for patients with depression. For example, although the FAsD has been shown to correlate strongly with the commonly used generic Brief Fatigue Inventory (BFI), there are important differences between the two measures in content validity. Whereas the BFI was designed for use in cancer patients [29], the FAsD was developed based on direct input of patients with depression as well as clinicians who treat depression [25]. As a result of this careful approach to establishing content validity, the FAsD items assess the specific types of fatigue and its impact that are likely to be experienced by patients with depression, and the items use words shared by patients during qualitative research. Therefore, unlike the generic instruments, the FAsD has established content validity in the target population, and the appropriate wording and content of the items for this specific population may lead to greater measurement precision. For example, the specific relevance of the items to this population led to the clear two-factor model based on a factor analysis conducted to derive FAsD subscales [25]. In this analysis, there was a clear distinction between items assessing experience and items assessing impact. Therefore, the FAsD allows for specific assessment of the impact of fatigue, in contrast to the BFI, which has been shown to yield only a global score supported by a strong single-factor model fit [29]. In sum, although there is clearly some overlap between the FAsD and generic instruments assessing fatigue, no other instrument has demonstrated content validity for the detailed assessment of this clinically important symptom and its impact among patients with depression [25].

The FAsD was developed following recommendations in the Food and Drug Administration PRO Guidance Document [31]. The items were initially drafted and refined based on literature review and qualitative research with clinicians and patients diagnosed with depression. Then, a psychometric validation study was conducted to identify subscales and examine reliability and validity of the measure. In this validation study, the FAsD demonstrated good factor structure, internal consistency reliability, test–retest reliability, and construct validity [25]. The purpose of the current study is to examine the questionnaire’s responsiveness to change and identify a responder definition that will assist with interpretation of treatment-related change.

Responsiveness is the extent to which a health status measure accurately detects change in a patient’s condition over time [32–34]. Demonstration of this measurement property is necessary for a PRO measure to be considered fit for the purpose of “identifying differences in scores over time in both individuals and groups who have changes with respect to the measured concept” [31]. Tests for responsiveness typically include effect size statistics as well as correlations of change scores with change in previously validated measures or indicators of the concept of interest. Responsiveness testing may also include comparison of change scores among patient subgroups categorized by an indicator of change in the relevant concept, such as patients’ or clinicians’ perceptions of change.

Once responsiveness has been demonstrated, establishing guidelines for the interpretation of PRO change scores can assist in recognizing when an important shift in patients’ health status has occurred. This step of instrument development was often characterized as identifying the minimally important difference (MID). However, the 2009 Food and Drug Administration (FDA) PRO Guidance has eliminated the term *MID* from their directives for PRO development.

Instead of the MID, the FDA now requests a *responder definition* that is “the individual patient PRO score change over a predetermined time period that should be interpreted as a treatment benefit” when a PRO instrument is used in clinical trials [31]. The FDA recommends that the responder definition should be determined empirically through anchor-based methods using data from the target population, with supportive evidence from distribution-based statistics. The anchors, which should be easier to interpret than the PRO measure, may be clinical indicators, patient ratings of change, or clinician ratings of change. Once a responder definition is ascertained, the percentage of responders achieving change at or beyond this threshold in each treatment arm of a clinical trial can be compared to facilitate the evaluation and communication of PRO results to patients, physicians, and providers.

## Methods

### Study design

Data were collected from patients with depression at seven privately owned psychiatry clinics specializing in behavioral and mental health in the United States. Inclusion criteria included: age ≥18 years old; clinical diagnosis of depression; and current symptoms of depression as indicated by a score on the 8-item Patient Health Questionnaire (PHQ-8) of ≥5, the recommended cutpoint for mild depression severity [35]. Patients were required to have started treatment with a new antidepressant within seven days prior to their first study visit. This treatment decision must have been made for clinically indicated reasons independent of the current study or any other study. Exclusion criteria were diagnosis of bipolar disorder; receiving treatment with a mood stabilizer or antipsychotic; or diagnosed with the following medical conditions that could cause fatigue: chronic fatigue syndrome, sleep apnea, cancer, multiple sclerosis, or HIV. Patients returned for a second visit six weeks after the initial study visit. The study protocol was approved by an independent ethics review committee (Ethical Review Committee, Inc.; ID#: 436-07-08), and all participants provided informed consent.

A total of 119 patients were enrolled. Patients were excluded from the current analysis if they did not attend Visit 2 (*n* = 18) or if they attended Visit 2 on a date that was outside the required window of 42 ± 7 days after Visit 1 (*n* = 5). Thus, there were 96 patients in the analysis sample.

### Measures

All measures described below were administered at both study visits unless stated otherwise.

#### Fatigue Associated with Depression Questionnaire (FAsD)

This 13-item patient-reported questionnaire was designed to assess fatigue associated with depression in the past week [25]. Three scores are computed: a 6-item fatigue experience subscale (items: fatigued, tired, exhausted, lack of energy, physically weak, and feeling like everything requires too much effort), a 7-item fatigue impact subscale (items assess impact on household chores; family relationships; enjoyable activities; social activities with friends; self-care; intimate relationships; and productivity at work or school), and a total score (all 13 items). Items 12 (impact on intimate relationships) and 13 (impact on productivity at work or school) are not applicable to all respondents, so these items are not answered in some cases. The fatigue experience items are rated on a 5-point scale with response options of “never,” “rarely,” “sometimes,” “often,” and “always.” The impact items are rated on a 5-point scale with response options of “not at all,” “a little,” “somewhat,” “quite a bit,” and “very much.” The two subscales and the total score are computed as the mean of all answered items within each scale, and each scale score has a possible range of 1 to 5, with higher scores representing greater fatigue.

#### Brief Fatigue Inventory

The brief fatigue inventory (BFI) includes three items assessing the severity of fatigue and six items assessing the degree to which fatigue has interfered with a range of domains, including mood, walking ability, and enjoyment of life [29]. The BFI was designed primarily for cancer patients, with a structure derived from the Brief Pain Inventory. The total score is computed as the mean of responses to all items. These scores may range from 0 to 10, with higher scores representing greater fatigue.

#### Epworth Sleepiness Scale

The Epworth Sleepiness Scale (ESS) [36] was developed to determine the level of daytime sleepiness, and it was administered in the current study so that analyses could explore the relationship between fatigue and sleepiness. Patients rate the chance of dozing or sleeping during eight activities. Scores are based on the sum of responses to the eight items. Scores may range from 0 to 24, with higher scores representing greater sleepiness.

#### Clinical Global Impression-Severity

The Clinical Global Impression-Severity Scale (CGI-S) was completed by clinicians to assess their overall impression of the severity of the patient’s depressive illness. The score ranges from 1 (normal, not at all ill) to 7 (among the most extremely ill patients) [37].

#### Patient perception of change

At Visit 2, patients reported their perceptions of change in fatigue. Patients were asked “Since you began your current antidepressant treatment about 6 weeks ago, has there been an overall change in your fatigue?” Patients responded by choosing one of seven response options: much worse; moderately worse; a little worse; stayed about the same; hardly any change; improved in a small but important way; moderately improved; and much improved.

#### Clinician perception of change

Clinicians completed two items at Visit 2. The first item asked clinicians to rate change in patients’ fatigue since beginning a new antidepressant treatment at the time of Visit 1. The second item asked whether there had been a change in the patient’s depression. Clinicians responded to both questions by choosing one of seven response options: much worse; moderately worse; a little worse; stayed about the same, hardly any change; improved in a small but important way; moderately improved; and much improved. Clinicians did not see patients’ responses to any questionnaires prior to completing these items.

#### Demographic and clinical forms

All participants completed a brief demographic and clinical form. Clinicians completed a clinical information form for each participant, reporting diagnoses, severity of depression, comorbid conditions, and medications.

### Statistical analysis

Descriptive statistics summarizing demographic and clinical characteristics were summarized in terms of frequencies and percentages for categorical variables as well as means and standard deviations for continuous variables.

#### Statistical procedures for assessing responsiveness of the FAsD

Clinician- and patient-rated measures were used to assess the responsiveness of the FAsD. Pearson correlations were performed to assess the degree of association between the FAsD change scores and change in the BFI and ESS. Correlation coefficient absolute values were interpreted as small (0.1 to 0.29), moderate (0.3 to 0.49), and large (0.5 and greater) based on the guidelines proposed by Cohen [38].

Patients were categorized into groups based on their degree of change as indicated by three variables: (1) change from Visit 1 to Visit 2 on the CGI-S, (2) clinician perceptions of change in fatigue, and (3) patient perceptions of change in fatigue. Then, the mean FAsD scores of these groups were compared using either *t* tests or general linear models (GLMs) with Scheffe’s post hoc pairwise comparisons, while controlling for age, gender, and antidepressant medication class. Medication class was a three-level categorical variable: selective serotonin reuptake inhibitor (SSRI), *n* = 51; serotonin–norepinephrine reuptake inhibitor (SNRI), *n* = 37; and other, *n* = 8. The eight patients in the “other” group were treated with either a norepinephrine–dopamine reuptake inhibitor (NDRI; *n* = 7) or a combination of an NDRI and an SSRI. The GLMs were also conducted using the BFI as the dependent variable to examine whether results were similar to those for the FAsD.

Effect size is a statistic that represents change as a standard unit of measurement [39]. For the subscales and total score of the FAsD, effect sizes were calculated as the difference in mean score from baseline to follow-up divided by the standard deviation of baseline scores for all subjects (mean score Time 1—mean score Time 2/standard deviation of baseline scores). This effect size was interpreted as small (0.20), moderate (0.50), or large (0.80) following the guidelines proposed by Cohen [38].

#### Statistical procedures for identifying the responder definition of the FAsD

The responder definition for each the FAsD subscales and total score was determined using anchor-based methods, supported by distribution-based approaches [31, 34]. The primary method used the mean change scores of patients who reported experiencing a small but important change in fatigue from Visit 1 to Visit 2. A similar secondary analysis was conducted using the anchor of clinician-reported change in fatigue. Beyond these anchor-based methods, the state changes for each FAsD subscale and total scores were considered when deriving the responder definitions. “State changes” are defined as the amount of change in a subscale or total score that results from one shift up or down in the response options for only one item [40].

Two distribution-based methods for examining responder definition were calculated using Visit 1 data. First, the standard error of measurement (SEM) was computed as the standard deviation of an observed score related to its reliability (SD * sqrt of [1—reliability]). The SEM has been linked to estimates of minimally important change standards [41]. The SEM is expressed in the original metric of the instrument, which can facilitate ease of interpretation. Although test–retest reliability is the most appropriate estimate of reliability for SEM calculations used to approximate an important change over time [42], internal consistency reliability was used for these analyses because reproducibility estimates could not be determined for the current sample. The second distribution-based method was the half standard deviation, which has been shown to provide a reasonable approximation of a meaningful change in patient-reported outcome instruments [43].

## Results

### Sample description

Table 1 presents demographic and clinical characteristics (*N* = 96). Participants were 55.2% women, with a mean age of 43.4 years. The sample was somewhat diverse with regard to racial background (e.g., 41.7% white, 27.1% African-American, and 16.7% Hispanic), marital status (e.g., 32.3% married, 32.3% single, 19.8% divorced), and employment status (e.g., 32.3% working full-time, 28.1% unemployed, 16.7% disabled, and 13.5% working part-time). The majority of the sample was living with a spouse, partner, family, or friends (78.1%) and had received at least some college education (65.7%). The mean age at the time of first depression diagnosis was 33.3 years. The majority of participants reported no comorbid medical conditions (61.5%). Among those reporting a comorbid medical condition, the most common conditions were hypertension (18.8%) and arthritis (11.5%). Based on medical chart review, a majority of the participants did not have any current or previous psychiatric diagnoses other than depression (81.3%). The most common current or previous comorbid psychiatric conditions were anxiety disorders (13.5%). At Visit 1, the majority of the sample was considered to have depression of at least moderate symptom severity, as rated by clinicians completing the CGI-S. These CGI-S ratings were as follows: borderline ill (*n* = 2; 2.2%), mildly ill (*n* = 4; 4.4%), moderately ill (*n* = 62; 68.1%), markedly ill (*n* = 21; 23.1%), and severely ill (*n* = 2; 2.2%).

**Table 1:** Demographic and clinical characteristics

Characteristic	Statistics (*N* = 96)
*Age* (mean, SD)	43.4 (11.4)
*Gender* (*n*, %)	
Male	43 (44.8%)
Female	53 (55.2%)
*Racial background* (*n*, %)	
Asian or Pacific Islander	9 (9.4%)
Black, not of Hispanic origin	26 (27.1%)
Hispanic	16 (16.7%)
White, not of Hispanic origin	40 (41.7%)
Other	5 (5.2%)
*Marital status* (*n*, %)	
Married	31 (32.3%)
Not married	65 (67.7%)
*Living/domestic situation* (*n*, %)	
Living alone	18 (18.8%)
Living with spouse, partner, family, friends	75 (78.1%)
Other	3 (3.1%)
*Employment status* (*n*, %)	
Full-time work	31 (32.3%)
Part-time work	13 (13.5%)
Other	52 (54.2%)
*Comorbid conditions* (*n*, %)	
Arthritis	11 (11.5%)
Diabetes	10 (10.4%)
Hypertension	18 (18.8%)
Other	18 (18.8%)
None	59 (61.5%)
*Antidepressant medications started within one week of Visit 1* (*n*, %)^a^	
NaSSA: (mirtazapine)	1 (1.0%)
NDRI: (bupropion)	7 (7.3%)
SNRI (desvenlafaxine, duloxetine, venlafaxine)	37 (38.5%)
SSRI (citalopram, escitalopram, fluoxetine, fluvoxamine, paroxetine, sertraline)	52 (54.2%)

^a^Of the 96 patients, 95 received only one new antidepressant, while one patient received combination treatment with two antidepressants (bupropion and citalopram)

*NaSSA* noradrenergic and specific serotonergic antidepressant/tetracyclic antidepressant

*NDRI* norepinephrine–dopamine reuptake inhibitor/aminoketone

*SNRI* serotonin–norepinephrine reuptake inhibitor

*SSRI* selective serotonin reuptake inhibitor or serotonin-specific reuptake inhibitor

All participants began treatment with a new antidepressant (Table 1) within seven days prior to their first study visit. The most commonly prescribed classes of medications were SSRIs (54.2%) and SNRIs (38.5%). The most frequent prescribed medications were escitalopram (30.2%), desvenlafaxine (15.6%), duloxetine (14.6%), and venlafaxine (8.3%).

#### Descriptive statistics: FAsD change

FAsD mean scores were lower at Visit 2 (total score = 2.84) than Visit 1 (total score = 3.53), indicating improvement in depression-related fatigue (Table 2). Mean score changes on the two FAsD subscales also reflected a decrease from Visit 1 to Visit 2 (decreases of 0.73 on the impact subscale and 0.67 on the experience subscale). The FAsD scales had moderate to large effect sizes from Visit 1 to Visit 2 (−0.76 for the impact subscale; −0.84 for the experience subscale; and −0.84 for the total score).

**Table 2:** FAsD change scores: *t* tests comparing Visit 1 score to Visit 2 score and correlations with change in other patient-reported measures

FAsD subscales	Visit 1 Mean (SD)	Visit 2 Mean (SD)	Change from Visit 1 to Visit 2 Mean (SD)	*t* value	Pearson correlations of FAsD change with change in other measures	
					BFI	ESS
FAsD experience subscale score	3.61 (0.80)	2.95 (0.96)	−0.67 (1.02)	−6.4***	0.73***	0.36***
FAsD impact subscale score	3.45 (0.95)	2.73 (1.16)	−0.73 (1.08)	−6.6***	0.73***	0.40***
FAsD total score	3.53 (0.83)	2.84 (1.01)	−0.69 (0.97)	−7.0***	0.80***	0.42***

*N* for means and *t* tests = 96; *N* for correlations with BFI = 95; *N* for correlations with ESS = 92

*FAsD* Fatigue Associated with Depression Questionnaire

*BFI* Brief Fatigue Inventory

*ESS* Epworth Sleepiness Scale

*** *p* < 0.001

Because the final two items of the FAsD impact scale are designed to be skipped by some patients for whom the items are not applicable (i.e., patients who do not attend work/school or have an intimate relationship), descriptive statistics were also conducted for the instrument without inclusion of these final two items. The mean impact subscale score was 3.47 at Visit 1 and 2.73 at Visit 2 (mean change score = −0.74). The mean total score was 3.55 at Visit 1 and 2.85 at Visit 2 (mean change score = −0.70). These values are almost the same as those presented in Table 2, which has scores computed with all 13 items.

#### Assessing responsiveness through comparisons to clinical measures of change

Changes in FAsD subscale and total scores were significantly (all *p* < 0.001) correlated with changes in the BFI and the ESS (Table 2). Correlations of the FAsD scales with the BFI ranged from 0.73 to 0.80. While still statistically significant, correlations with the ESS were weaker, ranging from 0.36 to 0.42.

FAsD mean change scores discriminated among groups of patients were categorized based on patient perceptions of change in fatigue (Table 3). In general, greater improvement in patient perceptions of fatigue was associated with greater improvement in the FAsD. For example, the FAsD total score and experience subscale score demonstrated significantly greater average change in the group that perceived improvements than in the groups that reported their fatigue as staying about the same or worsening (all *p* < 0.05). Analyses with patients categorized based on clinician-perceived change in fatigue yielded similar results. *T* tests were conducted to compare FAsD scores between patients who improved and those who did not improve (i.e., worsened or no change), based on clinician judgment. All FAsD scales demonstrated significantly greater change in patients who improved than in patients who did not improve (between-group differences were 0.53 for the experience subscale, 0.61 for the impact subscale, and 0.57 for the total score; all *p* < 0.05).

**Table 3:** Analysis of variance comparing FAsD and BFI change scores among groups differing by patient perception of change in fatigue

Change in FAsD scales	Three groups categorized based on patient perception of change in fatigue			Overall *F* value^a^	Patient perception of change in fatigue *p* value	Significant pairwise comparisons^b^
	Improved (*N* = 66) LS Mean (SEM)	About the same (*N* = 17) LS Mean (SEM)	Worsened (*N* = 10) LS Mean (SEM)			
FAsD experience subscale score	−0.96 (0.15)	−0.25 (0.25)	0.08 (0.32)	3.8**	<0.001	A*, B**
FAsD impact subscale score	−1.03 (0.16)	−0.45 (0.27)	−0.10 (0.35)	2.5*	0.011	B*
FAsD total score	−1.00 (0.14)	−0.34 (0.24)	−0.00 (0.30)	3.6**	<0.001	A*, B**
BFI	−2.78 (0.34)	−0.66 (0.58)	−0.34 (0.74)	3.8**	<0.001	A**, B**

^a^Model includes age, gender, and antidepressant medication class as covariates

^b^Pairwise comparisons: A = improved versus about the same; B = improved versus worsened

*FAsD* Fatigue Associated with Depression Questionnaire

*BFI* Brief Fatigue Inventory

* *p* < 0.05; ** *p* < 0.01

FAsD total and subscale mean change scores also significantly discriminated among groups of patients categorized based on degree of change in the clinician-rated CGI-S. Patients were categorized into three groups based on degree of CGI-S change: improved by 2 or 3 levels, improved by 1 level, and worsened/no change. Greater improvements in CGI-S ratings were associated with greater reduction in mean FAsD scores. For example, the FAsD total score decreased by 1.26 points among patients who improved by 2 or 3 CGI-S levels, 0.72 points among patients who improved by 1 CGI-S level, and −0.37 points among patients who did not improve in the CGI-S. Pairwise comparisons indicate that the FAsD impact subscale and total score demonstrated significantly greater average change in the improved by 2 or 3 levels group than in the no improvement group (all *p* < 0.05). The FAsD impact subscale also demonstrated significantly greater mean change in the improved by 2 or 3 levels group than in the improved by 1 level group (*p* < 0.05).

The ANOVA models were also conducted with the BFI, rather than the FAsD, as the dependent variable. The pattern of mean change scores and statistically significant differences between groups followed the same patterns as those resulting from models with the FAsD total score. Patient perception of greater improvement and greater improvement in CGI-S ratings were both associated with greater decreases in the BFI. As indicated in Table 3, the BFI demonstrated significantly greater change in the group that perceived improvements than in the groups that reported their fatigue as staying about the same or worsening (all *p* < 0.01).

#### Responder definition

The primary method for estimating responder definition was based on the mean FAsD change among the 20 patients who reported experiencing a small but important change in fatigue from the first study visit to the second (Table 4). The mean FAsD change scores for this subgroup were −0.66 (experience subscale), −0.51 (impact subscale), and −0.59 (total score). Analyses using clinician-reported anchors provide additional support for the responder definition (Table 4). Among the 17 patients viewed by clinicians as having a small but important change in fatigue, FAsD mean change scores were −0.37 (experience subscale), −0.25 (impact subscale), and −0.31 (total score). The smaller FAsD mean change scores corresponding to clinician-rated changes over time suggest that the patient-based change scores are a conservative estimate of the responder definition (i.e., the clinician-rated changes suggest that a smaller responder definition may be appropriate).

**Table 4:** Estimating the responder definition: FAsD change scores among groups of patients categorized based on change in fatigue

Perceptions of change in fatigue	N^a^ (%)	FAsD experience subscale (mean, SD)	FAsD impact subscale (mean, SD)	FAsD total score (mean, SD)
*Patient perception of change in fatigue* ^a^				
Much improved	16 (17.2%)	−1.41 (0.99)	−1.57 (1.09)	−1.50 (0.97)
Moderately improved	30 (32.3%)	−0.82 (1.01)	−0.88 (1.29)	−0.85 (1.09)
Improved in a small but important way	20 (21.5%)	−0.66 (0.91)	−0.51 (0.75)	−0.59 (0.63)
Stayed about the same	17 (18.3%)	−0.17 (0.78)	−0.32 (0.60)	−0.23 (0.62)
A little worse	5 (5.4%)	0.27 (0.76)	0.32 (0.36)	0.30 (0.39)
Moderately worse	3 (3.2%)	0.11 (1.02)	0.00 (0.40)	0.06 (0.64)
Much worse	2 (2.2%)	−0.17 (1.41)	−0.46 (0.56)	−0.33 (0.41)
*Clinician perception of change in fatigue* ^a^				
Much improved	15 (17.9%)	−1.24 (1.13)	−1.63 (1.16)	−1.44 (1.08)
Moderately improved	24 (28.6%)	−1.15 (1.02)	−1.25 (1.14)	−1.20 (1.02)
Improved in a small but important way	17 (20.2%)	−0.37 (0.78)	−0.25 (0.64)	−0.31 (0.56)
Stayed about the same	20 (23.8%)	−0.07 (0.74)	−0.25 (0.73)	−0.15 (0.58)
A little worse	6 (7.1%)	−0.44 (0.96)	0.14 (0.24)	−0.15 (0.49)
Moderately worse	1 (1.2%)	−1.17 (–)	−0.07 (–)	−0.62 (–)
Much worse	1 (1.2%)	−0.83 (–)	−0.14 (–)	−0.46 (–)

^a^Of 96 patients, 93 completed the patient perception of change question, and 84 had clinicians who reported perception of change in the patient’s fatigue

*FAsD* Fatigue Associated with Depression Questionnaire

Kappa statistics were calculated to examine the degree of agreement between patient perception of change in fatigue and clinician perception of change (the two variables used in Table 4) [44]. The simple kappa of 0.27 (95% confidence interval: 0.13 to 0.40) and the weighted Kappa of 0.49 (95% confidence interval: 0.38 to 0.61) suggest a small-to-moderate level of agreement between patient and clinician ratings.

Distribution-based methods also suggest that the patient-based change scores are a conservative estimate of responder definition. For the experience subscale, impact subscale, and total score, the half SD values were 0.40, 0.48 and 0.41, respectively. SEM values, using internal consistency reliability estimates from the current sample, were 0.30, 0.36 and 0.24.

The state change defined as the amount of change in a subscale, or total score that results from one shift up or down in the response options for only one item, was calculated for each FAsD subscale and the total scores. For the FAsD experience subscale with six items, a shift of one response option on a single item corresponds to a change of 0.17 in the subscale score. For the impact subscale with seven items, a shift of one response option on a single item corresponds to a change of 0.14 in the subscale score. Finally, the FAsD total score with 13 items has a state change of 0.08 points. Because the FAsD scores can only change by increments in multiples of these state changes, the responder definitions for the FAsD experience subscale, impact subscale, and total score were conservatively set at 0.67, 0.57 and 0.62, respectively.

## Discussion

Results of all analyses indicate that the FAsD was responsive to change. The total score and both subscales demonstrated statistically significant improvement from Visit 1 to Visit 2, with effect sizes suggesting that these changes were in the moderate to large range. In addition, FAsD change scores discriminated among groups of patients who differed by degree of improvement in patient- and clinician-reported fatigue and depression symptom severity.

Current results also provide an initial indication of a responder definition that may be used when interpreting treatment-related change in the FAsD. Using the 20 patients with ratings of a small but important improvement in fatigue after six weeks of treatment, the mean change scores in this study were −0.66, −0.51, and −0.59, respectively, for the FAsD experience subscale, impact subscale, and total score. These mean change scores are likely to be conservative estimates of a responder definition, because they exceed the magnitude of values provided by supportive analyses, including mean change scores among 17 patients viewed by clinicians as having a small but important change in fatigue, as well as the half standard deviation and the SEM of these scales. Based on these results and the magnitude of a state change in the FAsD experience subscale, a responder definition of 0.67 is recommended for this subscale (i.e., a score decrease of at least 0.67 on this subscale). This threshold corresponds to a shift of four response options across the six items in the subscale. Similarly, the responder definition for the FAsD impact subscale was identified as 0.57, which corresponds to a shift of four response options across the seven subscale items. Finally, the responder definition for the FAsD total score was identified as 0.62, which corresponds to a shift of eight response options across the 13 items.

The strong correlations between FAsD change scores and BFI change scores suggest that these two questionnaires capture change in similar aspects of fatigue. However, there are two key differences between the questionnaires. First, the FAsD was developed and validated specifically for patients with depression, suggesting that it may be uniquely fit for use in this target population. In contrast, the BFI was designed for use in cancer patients, with a general structure derived from the Brief Pain Inventory [29]. Second, the FAsD subscales provide separate assessments of fatigue experience and impact, whereas the BFI yields only a global score [29]. In qualitative research conducted when developing the FAsD, patients with depression have reported that fatigue has a powerful impact on multiple aspects of their lives [25], and the FAsD impact scale was designed to quantify this impact. Therefore, the FAsD has advantages over the BFI for studies examining change in fatigue among patients with depression. Furthermore, although correlations with the BFI are strong (0.73 ≤ *r* ≤ 0.80), these coefficients suggest that the BFI explains only 53% of the variation in the FAsD subscales and 64% of the variation in the FAsD total score. Therefore, the FAsD captures unique aspects of fatigue that are not captured by the BFI in this population.

One limitation of the current study is that patients received treatment in naturalistic clinical settings, rather than in a controlled clinical trial context. Although all patients were required to receive a new antidepressant treatment within 7 days of study enrollment, it is likely that many aspects of the treatment experience varied among the seven clinical sites, as well as among clinicians at each site. Therefore, the generalizability of the current results to the clinical trial context is not known. Another limitation is that the current sample size is not large enough to examine FAsD measurement properties within subgroups of patients categorized based on their specific pharmacological treatment. Patients in the current study received a wide range of pharmacological treatments. Some of these medications may have the potential to exacerbate fatigue, while others may have the potential to reduce fatigue, and it is possible that FAsD scores were influenced by these treatments. Nonetheless, these results support the use of the FAsD in studies examining change in fatigue, and the FAsD may be even more responsive to change in a controlled trial with a standardized treatment approach.

Another factor that could have affected the results is the missing data at Visit 2. Of the 119 patients who were enrolled in the study, 23 were excluded from the analyses either because they did not attend Visit 2 (*n* = 18) or they attended Visit 2 outside the required window of 42 ± 7 days after Visit 1 (*n* = 5). Because the goal of this analysis was to examine change in any instrument over time, it was essential to have data at a minimum of two time points. Therefore, no data were imputed for the missing Visit 2 values. It is possible that the 23 excluded patients could have had more severe symptoms or less improvement on average than the 96 included patients. However, although this potential difference between included and excluded patients could affect the evaluation of treatment outcomes, it is unlikely to have a substantial impact on the current analysis that focused on longitudinal instrument performance and ascertaining the responder definition to identify individuals with a treatment benefit. Because the 96 included patients demonstrated improvement in depression and fatigue, their data are likely to be sufficient for evaluation of FAsD responsiveness and responder definition.

In the current study, the responder definition was based primarily on patients who reported a small but important change. However, other methodological approaches are possible. For example, there may be situations when it is preferable or necessary to use clinicians’ ratings, rather than patients’ ratings, as the primary anchor of change [45]. Current results indicate that clinicians and patients may have different perspectives on meaningful change. In the current sample, 20 patients reported “small but important” change, compared with only 17 patients who had this rating of change from clinicians. The mean FAsD change scores were lower for the 17 patients classified to this change group by clinicians than for the 20 self-classified patients. These findings suggest that a clinician-based approach could yield a different responder definition than a patient-based approach. In addition, “small but important” may not be the optimal degree or description of change to select a responder. For some PRO instruments, perhaps a patient-reported “moderate improvement” response would be a more appropriate criterion for determining the responder definition.

Almost all results of the current analysis followed logical and expected patterns, with the exception of some FAsD change scores presented in Table 4. However, the unexpected results only occurred in the smaller groups who reported becoming worse during the study (*n* ≤ 6) and are likely to be a function of the small group sizes. All groups of larger size (i.e., 16–30 patients categorized based on patient perception; 15–24 patients categorized based on clinician perception) followed logical patterns, with FAsD change scores of direction and magnitude that were entirely consistent with patient-reported and clinician-reported perception of change in fatigue. Future research with larger sample sizes may provide stronger support for the use of the FAsD to assess change over time.

When considered along with previous analyses demonstrating factor structure, reliability, and validity of the FAsD, current findings suggest that the FAsD is a useful measure of fatigue for studies focusing on treatment of depression. Measures such as the FAsD that allow for a detailed assessment of individual depressive symptoms are essential tools for developing a symptom-specific approach to treatment [3, 4]. By administering symptom-specific PRO measures, researchers may examine the effects of medications and other interventions on individual symptoms that are particularly relevant for some patients.
